# Changes in Cognitive Outcomes in Early Childhood: The Role of Family Income and Volatility

**DOI:** 10.3389/fpsyg.2022.758082

**Published:** 2022-03-14

**Authors:** Edward M. Sosu, Peter Schmidt

**Affiliations:** ^1^School of Education, Faculty of Humanities & Social Sciences, University of Strathclyde, Glasgow, United Kingdom; ^2^Department of Psychosomatic Medicine and Psychotherapy, University Medical Centre, Johannes Gutenberg University Mainz, Mainz, Germany; ^3^Center for International Development and Environmental Research, University of Giessen, Giessen, Germany

**Keywords:** family income, income volatility, cognitive development, early childhood, poverty, income dynamics, family stress, family investment

## Abstract

Associations between family income and child developmental outcomes are well documented. However, family income is not static but changes over time. Although this volatility represents income shocks that are likely to affect children’s lives, very few studies have so far examined its effect on early cognitive development. This study investigated associations between family income, volatility, and changes in cognitive outcomes in early childhood and examined whether these associations are dependent on a family’s overall income position. Data for the study spanned five waves of the Growing Up in Scotland longitudinal survey (*N* = 3,621). Findings indicate that income volatility was more prevalent among disadvantaged sociodemographic groups. In addition to average income, short-term volatility was associated with changes in child cognitive outcomes from ages 3 to 5. While upward volatility was associated with gains in expressive vocabulary, downward and fluctuating volatility were associated with declines in child problem-solving abilities. The association between volatility and changes in cognitive outcomes was similar for both children living in poverty and those from medium–high-income households. Our results suggest that policies aiming to cushion all families from negative income shocks, boost family income to ensure stability, and take low-income families out of poverty will have a significant impact on children’s cognitive development. Additionally, a more nuanced conceptualization of income is needed to understand its multidimensional impact on developmental outcomes.

## Introduction

Strong associations exist between family income and cognitive development, with children from low-income households having significantly lower cognitive outcomes compared to peers from affluent backgrounds (Yeung et al., 2002; Dickerson and Popli, 2016; Cooper and Stewart, 2021). This early inequality in cognitive ability determines educational, health, and labor market outcomes in adulthood (Carneiro et al., 2007; Schoon, 2010; Henderson et al., 2012; Hardy, 2014), and accounts for the intergenerational transmission of disadvantages (Serafino and Tonkin, 2014). However, several complexities exist in the association between family income and child cognitive outcomes. Specifically, family income is not static but changes over time and this volatility represents income shocks that are likely to affect children’s lives (Bartels and Bönke, 2013; Bøe et al., 2017; Miller and Votruba-Drzal, 2017; Hill, 2018). Although, emerging evidence suggests that family income volatility may affect child developmental outcomes over and above-average income levels (e.g., Hardy, 2014; Miller and Votruba-Drzal, 2017; Gennetian et al., 2018), very few studies have so far examined its effect on early cognitive development. Most studies continue to use average or time-specific income to predict cognitive outcomes across the life course. This lack of nuance in how family income is conceptualized is problematic because it misses the effect of other dynamics of income on developmental outcomes and does not fully capture all individuals who may experience economic hardship (Bradshaw and Finch, 2003; Dynan et al., 2012).

The way in which volatility influences developmental outcomes may also depend on the form of volatility (i.e., upward, downward, or fluctuating) as well as a family’s overall economic condition, an important dimension that is not fully examined in current developmental research (Hardy, 2014; Gennetian et al., 2018; Hill, 2018). Finally, despite the assumption that changes in children’s typical experiences lead to changes in child outcomes, we are not aware of any developmental studies that have empirically examined how different forms of volatility in family income influences changes in developmental outcomes, especially in the early years.

The current study aims to address these gaps by examining the association between family income, volatility, and changes in cognitive outcomes in early childhood. It will also examine whether these associations are dependent on a family’s overall income position. Our study is particularly timely given the economic instability initiated by the more recent COVID-19 lockdown as the period we examine in this study (2005–2010) covers the Great Recession (2008–2012), a period equally characterized by significant instability in household earnings (Avram et al., 2019). In other words, findings from this study will provide insight into the possible impact of household income volatility arising from the COVID-19 pandemic on child cognitive outcomes, and lessons on how to attenuate possible negative future effects.

### Family Income Dynamics and Cognitive Development in Early Childhood

Evidence of substantial increases in household income instability over time has led to calls for a shift from the predominant conceptualization of family income as a static construct to include dynamic aspects such as volatility (Dynan et al., 2012; Hill, 2018; Avram et al., 2019). Income volatility is defined as the degree to which families experience sizeable swings in income over time (Dynan et al., 2012; Hill, 2018). It may occur because of changes in employment (Hollister and Smith, 2014), family relationships (Cherlin, 2010), or housing (Adam and Chase-Lansdale, 2002; Desmond and Shollenberger, 2015), and it disproportionately affects disadvantaged groups (Hill, 2018). Volatility is predicted to rise in the coming years due to the global COVID-19 pandemic which has resulted in significant changes in employment and job losses around the world (Bourquin et al., 2020; Kansiime et al., 2021).

According to Dynan et al. (2012), over and above income levels, volatility of income additionally affects families, and consequently child developmental outcomes. This is because volatility of household earnings is crucial for economic decision-making. Although income volatility, overall, is detrimental to the wellbeing of families, not all forms of volatility are negative (Hill, 2018). Volatility can take the form of income gain (upward volatility), income loss (downward volatility), or fluctuation (rise followed by fall or vice versa), and these dynamics might have positive or negative effects on child developmental outcomes.

Developmental theories provide a broad framework for understanding the association between family income dynamics such as volatility and child cognitive outcomes. The *bioecological model* (Bronfenbrenner, 1994) proposes that life course outcomes, such as cognitive development, are influenced by complex bidirectional interactions between the child and his or her proximal (e.g., family, school, and peers) and distal environments (e.g., government agencies and policies, location, and culture). Family income dynamics determine cognitive development by shaping children’s dispositions, their proximal and distal environments, as well as the way they interact with these environments. The exact social mechanisms (Hedström and Swedberg, 1998; Opp, 2005) by which dynamics of income influence cognitive development are explained by dominant theoretical perspectives such as family investment (Mayer, 1997; Conger et al., 2010) and stress models (Conger et al., 2010).

According to the *investment model* (Mayer, 1997, 2002; Shaw and Shelleby, 2014), family income influences child developmental outcomes by determining parents’ ability to invest in educational resources, good nutrition, health, housing, neighborhood, and other inputs that are crucial for children’s cognitive development (Mayer, 1997, 2002; Wolf and McCoy, 2019). Those with higher income are able to invest in these goods and services, and this leads to higher cognitive outcomes for their children, while families on lower incomes are restricted in their ability to invest resulting in lower cognitive outcomes for their children (Shaw and Shelleby, 2014). The *family stress model* on the other hand (Conger et al., 2010; Shaw and Shelleby, 2014) suggests that familial economic circumstances have an indirect effect on child outcomes through parental psychological health and behaviors. Economic pressures and the struggle of having fewer resources to pay for day-to-day essentials leads to significant psychological distress among parents. These increased psychological stressors in turn lead to greater family conflict, household chaos, and negative parent–child relationships, such as the use of unresponsive parenting styles that are detrimental to children’s behavioral and cognitive outcomes (Kiernan and Huerta, 2008; Rijlaarsdam et al., 2013; Sosu and Schmidt, 2017; Seidler and Ritchie, 2018; Baker et al., 2021). Apart from the effect on parents, economic pressures can lead to greater levels of stress and allostatic load in children, and these factors in turn have negative consequences for cognitive development (Evans et al., 2007; Blair et al., 2011; Miller et al., 2011; Ursache et al., 2015).

Drawing on the family stress and investment models, we hypothesize that upward volatility in the form of income gains will have positive effects on developmental outcomes by endowing parents with the capacity to invest in the goods and services that improve cognitive outcomes for children (e.g., Akee et al., 2010; Duncan et al., 2011; Bastagli et al., 2018). Conversely, downward volatility will reduce familial resources and ability to afford the things that support child cognitive development, leading to negative outcomes (e.g., Dahl et al., 2014). Additionally, the family stress model suggests that gains should lead to better parental psychological outcomes and positive parent–child relationships that benefit cognitive outcomes, while losses will harm parental mental health and lead to negative parent–child relationships that are detrimental to cognitive development (Prause et al., 2009; Miller and Votruba-Drzal, 2017; Cooper and Stewart, 2021). Income fluctuation, which represents an increase in income followed by a fall or vice versa should have an overall negative effect due to uncertainties in the level and timing of parental investments, greater parental stressors, poor mental health, and negative parent–child relationships (Prause et al., 2009; Hardy, 2014; Hill, 2018).

While there is extensive evidence on the association between family income and child cognitive development (see e.g., Sirin, 2005; Cooper and Stewart, 2021), research examining the consequences of income volatility on early cognitive outcomes is generally lacking. The only study we found that examined income volatility and cognitive ability in early childhood reported a non-significant effect of 30% downward volatility on cognitive outcomes (Yeung et al., 2002). Another study on cognitive outcomes examined the impact of volatility on postsecondary educational attainment (Hardy, 2014). It found that experiences of 25% income volatility in childhood had a “modest effect” on attainment in adulthood, with permanent income having the strongest impact. Studies that have examined association between volatility and other developmental outcomes from mid-childhood to early adolescence have found mixed associations. For instance, Miller and Votruba-Drzal (2017) found a significant association between downward volatility of 20% and a trajectory of externalizing and internalizing behavioral problems from mid-childhood to late childhood. Contrary to their hypothesis, there were no associations between upward volatility and these behavioral outcomes. Gennetian et al. (2018) found that high levels of overall income volatility were associated with lower school attendance among 9- to 12-year-olds. Finally, Gennetian et al. (2015) found that the number of intra-year income shocks was associated with lower adolescent school engagement, although these relationships were not significant when looking at 33% upward or downward volatility. The variability in these findings raises questions about the relative importance of income volatility for cognitive development in early childhood.

One key proposition from existing studies is that the effect of volatility on developmental outcomes may depend on a family’s overall income position (e.g., Hardy, 2014; Gennetian et al., 2015). Shocks are more likely to affect those with lower income due to absence of savings to deal with such short-term shocks (Page et al., 2009; Hardy, 2014). Higher earners, on the other hand, may have enough reserves to cushion such shocks. Empirical studies on young adults in the United States found mixed evidence in support of this proposition (Hardy, 2014; Gennetian et al., 2015). For instance, Hardy (2014) found that the association between volatility and postsecondary attainment was largest for those from moderate compared to low- and high-income households. However, this association was not statistically significant at the conventional rate. Gennetian et al. (2015), on the other hand, found that volatility was statistically related to school expulsion for adolescents from the lowest income but not for higher income quintile households, consistent with their hypothesis. For school engagement, they found that the volatility effect holds for adolescents from the first and fifth income quintiles but not for those from middle-income quintiles. We are not aware of studies that have looked at this claim in younger children.

### Aims of the Current Study

The current study aims to extend existing theory and empirical evidence on family income conditions and developmental outcomes by addressing existing gaps in research on income volatility and developmental outcomes of children.

First, most existing studies on family income volatility and developmental outcomes have mainly examined the effects of upward and downward volatility (e.g., Hardy, 2014). Although reference has been made to “fluctuating volatility” or “economic instability,” which refers to a combination of income gain and loss or vice versa, no research has so far examined the effect of this conceptualization on childhood outcomes (Hill, 2018; Latner, 2018).

Second, the vast majority of the studies on income volatility and life course outcomes are from a United States context (e.g., Yeung et al., 2002; Hardy, 2014; Gennetian et al., 2015, 2018; Miller and Votruba-Drzal, 2017). While these provide important baseline evidence, the effect of volatility on developmental outcomes in other contexts such as the United Kingdom or Europe may differ due to availability of greater social support (Bartels and Bönke, 2013), that protects against income volatility especially for those living in poverty (Avram et al., 2019). Research within these contexts of relatively greater social support systems may provide new information on the comparative influence of volatility on child outcomes and policy mechanisms for addressing its consequences.

Finally, despite the assumption that changes in children’s typical experiences lead to changes in child outcomes, no studies have empirically examined how forms of volatility in income influences changes in cognitive outcomes, especially in the early childhood years.

We address these gaps by examining associations between family income, volatility, and changes in cognitive outcomes in early childhood. The following research questions have been formulated to achieve these goals:

1. What are the associations between family income and volatility and child cognitive development?
  - To what extent is family average income and income volatility associated with changes in child cognitive outcomes from ages 3 to 5.
  - Are the various forms of income volatility (upward, downward, and fluctuating) associated with changes in cognitive ability over and above-average income?
2. Do associations between family income volatility and changes in child cognitive development depend on a family’s overall income position?

## Materials and Methods

### Data and Sample

This study drew on data from the Growing Up in Scotland (GUS) longitudinal survey. The GUS uses multi-stage stratified random sampling to select eligible children to achieve a nationally representative sample. Face-to-face interviews were undertaken with a cohort child’s main carer (mostly mothers, 95.5%) annually (see ScotCen Social Research, 2013, for a detailed description).

For this study, we used data from wave 1 (obtained in 2005/06) to wave 5 (2009/10) of the first Birth Cohort survey. Data for the first wave were obtained when the cohort children were 10.5 months old, with subsequent waves taking place when they were aged 22, 34.5, 46, and 58 months, respectively. A total of 3,621 participants who responded to all five waves and for whom a longitudinal weight exists were retained for analysis. This represents 69.4% of all wave 1 participants. Attrition analysis suggests that missing data was common among those who were unemployed, lived in urban areas, were less likely to indicate their income, or younger parents (ScotCen Social Research, 2013). Potential biases of non-random sample attrition were addressed by using the longitudinal weights generated using characteristics associated with non-response in the analysis (ScotCen Social Research, 2013; Sosu and Schmidt, 2017). Of the total sample, 51% were male, with the majority of participants (96%) being of “White” ethnic origin.

### Ethical Approval

Ethical approval for the GUS study was received from Scotland’s Scottish “A” Multicentre Research Ethics Committee (MREC; application reference: 04/M RE 1 0/59), and approval for the data use for this study was obtained through the United Kingdom Data Service.

### Measures

#### Child Cognitive Ability

Cognitive ability was measured at about age 3 (34.5 months) and 5 (58 months), using the Naming Vocabulary and Picture Similarities subtests of the British Ability Scales Second Edition (BAS II). Previous analysis using these two scales from the GUS data suggests they are valid and reliable (Sosu and Schmidt, 2017). The Naming Vocabulary subscale assesses expressive language ability and development, while the Picture Similarities subscale assesses problem-solving and reasoning ability. T-scores derived from normative scores (with a range from 20 to 80, and a mean of 50) for both scales were used for analysis. There were moderate correlations between subscales (age 3 *r* = 0.46; age 5 *r* = 0.37) and between similar domains across time (naming vocabulary *r* = 0.56; picture similarity *r* = 0.32).

#### Forms of Income Dynamics

Three forms of income dynamics—*average family income*, *income volatility,* and *overall income position* (poverty status)—were derived from measures of equivalized income. Family equivalized income was measured across all five waves of data collection. Participants were first asked to select from a range of 17 income bands per year (1: less than £3,999 to 17: £56,000 or more), the amount which best represents their family income before tax including all state benefits and interests. All income bands between the minimum and maximum had a range of about £2,000 (i.e., £4,000–£5,999; £6,000–£7,999). The figures were equivalized by adjusting for differences in household size and composition to generate equivalized household incomes in the GUS survey (see e.g., Scottish Government, 2009; Bradshaw et al., 2015).

In order to reduce the probability of biased estimates associated with missing data, we addressed the problem of missing income across waves prior to computing the different forms of income dynamics. Specifically, we applied multiple imputation using the Markov Chain Monte Carlo (MCMC) method in the SPSS Version 25 software to estimate missing income at each wave. Given that the proportion of missing data of the income data were generally small (below 10%) a total of five imputations were estimated and we subsequently used robust full information maximum likelihood estimation for our main analysis to address missing data on other predictor and outcome variables (Schafer and Olsen, 1998; Graham et al., 2007). All imputations were undertaken using key sociodemographic independent variables included in our final model (e.g., maternal age at birth of cohort child, parental education, class, neighborhood deprivation, and family type). Imputed incomes were constrained within the absolute minimum and maximum values at each wave. The imputed income from all five data sets at each wave were pooled, and the average was used to replace the missing income for that wave. The proportion of missing income across each wave was less than 10% (Wave 1–9%; Wave 2–4.8%; Wave 3–6.2%; Wave 4–4.9%; Wave 5–5.7%). There were no statistical significant differences in average income between non-imputed and imputed data across Wave 1 [*t*(6,914) = −0.025, *p* = 0.98], Wave 2 [*t*(7,067) = 0.158, *p* = 0.88], Wave 3 [*t*(7,016) = 0.261, *p* = 0.79], Wave 4 [*t*(7,061) = 0.362, *p* = 0.72], and Wave 5 [*t*(7,034) = 0.584, *p* = 0.56].

##### Average Family Income

To measure average family income, we computed the mean for equivalized income across all five waves. The average family income level was £21,590 (ranging from £2,402 to £55,976; SD = £11,385). The average income level across each of the five waves indicates increasing income across time: [*M1* = £21,882 (SD = £12,389); *M2* = £23,161 (SD = £12,745); *M3* = £24,008 (SD = £12,443); *M4* = £24,476 (SD = £12,211); *M5* = £24,573 (SD = £12,247)].

##### Income Volatility

Income volatility was measured using the standard deviation of Arc Percentage Change (APC). The APC measures the average difference in income between two time points relative to the mean value across the two time points (Dynan et al., 2012; Gennetian et al., 2019). Compared to other approaches, the APC has the advantage of being symmetric regarding increases and decreases in income. It is bounded by −200 and 200 and can be intuitively interpreted as a percentage change (Dynan et al., 2012). The APC can also be applied equivalently for different subgroups, and estimates can be computed for an income value of zero as it is not dependent on starting values (Dynan et al., 2012; Gennetian et al., 2019). Finally, alternative approaches to computing volatility, such as the coefficient of variation and transitory income, tend to produce similar findings (Gennetian et al., 2018). We used income measured in waves 3–5 to compute volatility, as we were interested in estimating the degree to which family income volatility is associated with changes in children’s cognitive outcomes from age 3 (wave 3) to 5 (wave 5).

To estimate volatility for each participant, we first calculated the percentage change in income between every consecutive year (waves 3 and 4; waves 4 and 5) as:


$$\left[\left(Y_{t2}-Y_{t1}\right)/0.5\;\left(Y_{t1}+Y_{t2}\right)\right]\times 100$$

where *Y* = income and *t* = time. Second, because volatility captures different forms of income changes (negative, positive, or fluctuations), we created separate measures of income volatility using the conventional threshold of ≥25% income change (Gennetian et al., 2019; Miller et al., 2021). We did not adjust for income inflation because inflation alone could result in 25% income change for some categories of individuals but not others (Elfassy et al., 2019). Those who experienced gains at both consecutive years (waves 3–4; waves 4–5), or a single gain and no change in any of the periods, were classified as experiencing *Upward Volatility*. Those who experienced two losses, or a single loss and no change, were classified as having experienced *Downward Volatility*. Finally, we created a third indicator of *Fluctuating Volatility* to signify individuals who experienced either a gain followed by a loss, or vice versa. We did not examine the number of changes in upward and downward volatility as sample sizes were relatively small in some categories. All groups were referenced against individuals who experienced no change.

##### Overall Income Position

Overall income position was a dichotomous variable derived from average family income from wave 1 to wave 5. This was computed by using the poverty threshold in Scotland in 2009 when wave 5 data was collected (Scottish Government, 2011). Specifically, families whose average income across the five waves was below 60% of the national median income in 2009 (£13,072), indicative of those in poverty, were classified as low income (1) while those with income above this threshold were classified as medium–high income (0).

#### Covariates

To estimate the unique association between dynamics of income and cognitive development, we controlled for a rich set of child and parental sociodemographic characteristics known to influence family income dynamics and child cognitive ability (see Table 1). Child variables include *gender* (male and female) and *age* in months at wave 5. Parental variables include *mother’s age at birth of cohort* child; *family type* which measured whether the child was in a single or two parent household; *parental education* measured using the educational level of the parent with the highest qualification; *parental class* using the three-class National Statistics Socioeconomic classification (NS-SEC) of the parent with the highest class category (Office for National Statistics (ONS), 2016); and *neighborhood deprivation* measured using the Scottish Index of Multiple Deprivation (SIMD; Scottish Government, 2016).

**Table 1:** Descriptive statistics of variables in analysis.

	*N*^1^	Mean (*SD*)/Proportion	Min–Max
Naming vocabulary (age 3)	3,416	52.98 (*12.25*)	20–80
Naming vocabulary (age 5)	3,521	59.41 (*10.42*)	20–80
Picture similarity (age 3)	3,431	50.53 (*10.32*)	23–80
Picture similarity (age 5)	3,518	58.90 (*10.50*)	20–80
Income (British £, T1–T5)^2^	3,621	21,590 (*11,385*)	2,402–55,976
Income volatility (%)^2^	3,621	–	0–1
Stable income	1,856	46.1%	–
Upward (gaining)	738	22.5%	–
Downward (losing)	591	17%	–
Fluctuating (↓↑/↑↓)	436	14.4%	–
Child gender (%)	3,621	–	0–1
Female	1,767	51.2%	–
Male	1,854	48.8%	–
Child age at wave 5 (months)	3,621	58.15 (0.45)	57–60
Mother age at birth of cohort child (%)	3,611	–	0–1
>30	2,146	59.5	–
20–29	1,305	36.1	–
<20	160	4.4	–
Neighborhood poverty (SIMD, %)	3,621	–	–
SIMD 5 (least deprived)	787	21.7%	–
SIMD 4	785	21.7%	–
SIMD 3	780	21.5%	–
SIMD 2	621	17.1%	–
SIMD 1 (most deprived)	648	17.9%	–
Family type	3,621	–	0–1
Couple	3,131	86.5%	–
Single	490	13.5%	–
Parental education	3,618	–	0–1
Degree	675	18.7%	–
Vocational	1,308	36.2%	–
High school	1,170	32.3%	–
No qualification	465	12.9%	–
Parental class	3,621	–	0–1
Higher manag/admin/prof	946	26.1%	–
Intermediate	847	23.4%	–
Routine/manual/No work	1,828	50.5%	–

SIMD, Scottish Index of Multiple Deprivation; Higher manag/admin/prof, higher managerial, administrative, and professional occupations.

1 *N* is based on unweighted sample.

2 Mean (standard deviation) and proportion are computed using longitudinal weighted sample.

### Analysis Procedures

To test our first research questions (1a and 1b), we used a regression model with directly observed variables to estimate the association between income (*X*1) and volatility (*X*2) on the one hand, and domain-specific measures of cognitive outcomes (*Y*1, *Y*2) given a set of covariates (*Z*). We chose this approach because of the moderate correlation between the two cognitive measures. To ensure that our models estimate impact of income on *change in cognitive ability*, we employed residualized change models, whereby age 5 cognitive outcomes were included as dependent variables (*Y*1*_t1_*, *Y*2*_t1_*) while controlling for age 3 cognitive outcomes (*X*3, *X*4; Blair et al., 2014). The two basic regression equations were therefore:


(1) $$\begin{array}{l} Y1_{t1}=c_{t1}+\beta_{yx1}*X1_{t0}+\beta_{yx2}*X2_{t0}+\\ \;\;\;\;\;\;\;\;\;\;\;\;\;\;\;\beta_{yx3}*X3_{t0}+\beta *Z+e_{1} \end{array}$$

(2) $$\begin{array}{l} Y2_{t1}=c_{t1}+\beta_{yx1}*X1_{t0}+\beta_{yx2}*X2_{t0}+\\ \;\;\;\;\;\;\;\;\;\;\;\;\;\;\;\;\beta_{yx4}*X4_{t0}+\beta *Z+e_{2} \end{array}$$

where *Y*1_t1_ = Naming Vocabulary, *Y*2_t1_ = Picture Similarities, *c* = constant and *e* = error term, *X*1 = income, *X*2 = volatility, X3 and X4 = age 3 cognitive outcomes, and *Z* = covariates.

Adjustment for age 3 cognitive outcome also helps to control for possible unobserved variable bias in our analysis.

To examine our second research question examining the extent to which association between volatility and child cognitive outcomes is moderated by overall household income positions, we undertook a multigroup regression analysis. This implied estimating the association between dynamics of income and changes in cognitive outcomes for children who were living in poverty (*g*1) and those from medium–high-income households (*g*2) taking into account the whole set of control variables *Z*. We also constrained these associations to be equal across both groups. To test the plausibility of our assumption, we examined whether there was a significant difference in the log-likelihoods between our specified model and one without these constraints. The corresponding equations are:

Poverty households (*g*1)


(3) $$\begin{array}{l} Y1_{t1}^{g1}=c_{t1}^{g1}+\beta_{yx1}^{g1}*X1_{t0}^{g1}+\beta_{yx2}^{g1}*X2_{t0}^{g1}+\\ \;\;\;\;\;\;\;\;\;\;\;\;\;\;\;\;\;\beta_{yx3}^{g1}*X3_{t0}^{g1}+\beta^{g1}*Z^{g1}+e_{1}^{g1} \end{array}$$

(4) $$\begin{array}{l} Y2_{t1}^{g1}=c_{t1}^{g1}+\beta_{yx1}^{g1}*X1_{t0}^{g1}+\beta_{yx2}^{g1}*X2_{t0}^{g1}+\\ \;\;\;\;\;\;\;\;\;\;\;\;\;\;\;\;\;\beta_{yx4}^{g1}*X4_{t0}^{g1}+\beta^{g1}*Z^{g1}+e_{2}^{g1} \end{array}$$

Medium-/high-income households (*g*2)


(5) $$\begin{array}{l} Y1_{t1}^{g2}=c_{t1}^{g2}+\beta_{yx1}^{g2}*X1_{t0}^{g2}+\beta_{yx2}^{g2}*X2_{t0}^{g2}+\\ \;\;\;\;\;\;\;\;\;\;\;\;\;\;\;\;\beta_{yx3}^{g2}*X3_{t0}^{g2}+\beta^{g2}*Z^{g2}+e_{t1}^{g2} \end{array}$$

(6) $$\begin{array}{l} Y2_{t1}^{g2}=c_{t1}^{g2}+\beta_{yx1}^{g2}*X1_{t0}^{g2}+\beta_{yx2}^{g2}*X2_{t0}^{g2}+\\ \;\;\;\;\;\;\;\;\;\;\;\;\;\;\;\;\;\;\beta_{yx4}^{g2}*X4_{t0}^{g2}+\beta^{g2}*Z^{g2}+e_{t1}^{g2} \end{array}$$

where models (3) and (5), and (4) and (6) are constrained to be equal (3 = 5; 4 = 6).

Data were analyzed using Mplus 8.7 (Muthén and Muthén, 2012) with robust full information maximum likelihood estimation (RML) to take into account violation of the assumption of multivariate normality. We accounted for the study design complexity by taking into account the longitudinal weights, stratification, and data clusters.

## Results

### Descriptive: Family Profile and Income Volatility

**Figure 1:**
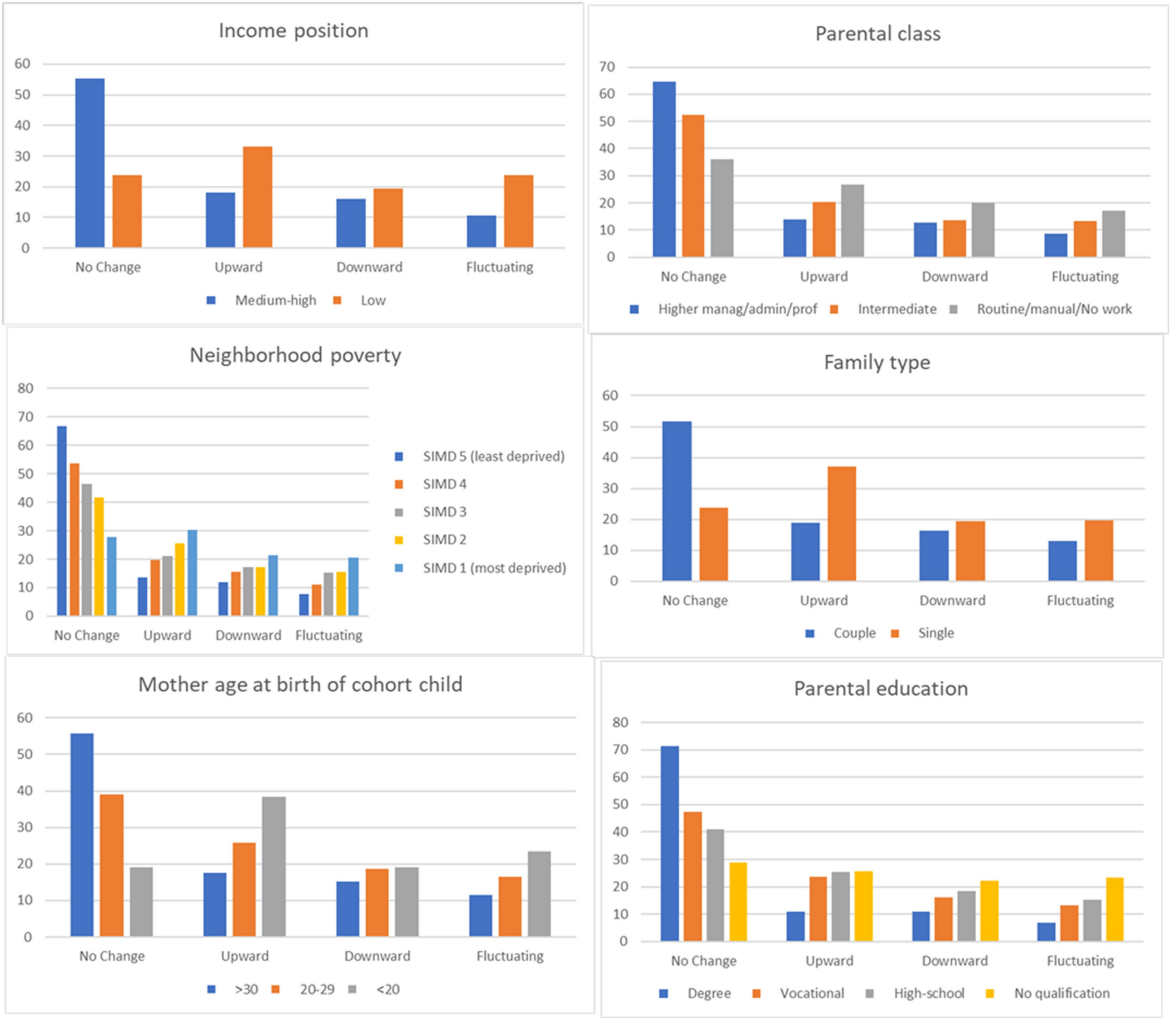
Social demographic profiles of families by forms of income volatility (%).

The descriptive characteristics of measures are provided in Table 1. Figure 1 shows the distribution of sociodemographic groups across the different income volatility categories. Families with an overall medium–high income, higher education, economic class, those living in affluent neighborhoods, from two parent households, and older mothers were more likely to experience income stability. Income volatility (upward, downward, and fluctuating) was greater for families in poverty, those with lower levels of education, socioeconomic class, those living in more deprived neighborhoods, single parents, and younger mothers. Results from bivariate multinomial regression (Table 2) indicate that these family sociodemographic characteristics were significantly associated with income volatility.

**Table 2:** Bivariate association between family socioeconomic characteristics and forms of income volatility.

	No change (reference category)		
	Upward	Downward	Fluctuating
	*B* (SE)	*B* (SE)	*B* (SE)
Income position (T1–T5)			
Medium–high	−1.44 (0.10)^***^	−1.04 (0.11)^***^	−1.65 (0.11)^***^
Low (Ref)	–	–	–
Parental education			
Degree	−1.75 (0.18)^***^	−1.61 (0.18)^***^	−2.13 (0.20)^***^
Vocational	−0.58 (0.13)^***^	−0.80 (0.14)^***^	−1.07 (0.15)^***^
High school	−0.37 (0.13)^***^	−0.54 (0.14)^***^	−0.78 (0.14)^***^
No qualification	–	–	–
Parental class			
Higher manag/admin/prof	−1.24 (0.12)^***^	−1.04 (0.12)^***^	−1.27 (0.14)^***^
Intermediate	−0.65(0.11)^***^	−0.76 (0.13)^***^	−0.62 (0.13)^***^
Routine/manual/No work	–	–	–
Neighborhood poverty			
SIMD 5 (least deprived)	−1.68 (0.15)^***^	−1.46 (0.16)^***^	−1.85 (0.18)^***^
SIMD 4	−1.09 (0.13)^***^	−0.98 (0.15)^***^	−1.26 (0.16)^***^
SIMD 3	−0.87 (0.13)^***^	−0.73 (0.14)^***^	−0.82 (0.15)^***^
SIMD 2	−0.57 (0.13)^***^	−0.63 (0.15)^***^	−0.68 (0.15)^***^
SIMD 1 (most deprived)	–	–	–
Family type			
Couple	−1.45 (0.11)^***^	−0.95 (0.13)^***^	−1.20 (0.13)^***^
Single	–	–	–
Mother age at birth of cohort child			
>30	−1.86 (0.18)^***^	−1.32 (0.21)^***^	−1.80 (0.20)^***^
20–29	−1.12 (0.18)^***^	−0.75 (0.21)^***^	−1.08 (0.20)^***^
<20	–	–	–

SIMD, Scottish Index of Multiple Deprivation; Higher manag/admin/prof, higher managerial, administrative, and professional occupations. Statistical significant association was tested using bivariate multinomial logistic regression.***p < 0.001.

### Association Between Family Income and Volatility, and Child Cognitive Development

Table 3 reports findings examining the association between family income, volatility, and changes in child cognitive outcomes measures. With respect to naming vocabulary, both average income (*β* = 0.081, *p* < 0.001) and income gain (*β* = 0.036, *p* < 0.05) were significantly associated with changes in naming vocabulary outcomes from ages 3 to 5. Specifically, children from households with higher average income recorded significant increases in receptive vocabulary scores. Compared to children from stable income households, those from households that experienced upward income demonstrated significantly larger growth in naming vocabulary scores across time, although children from stable income households had higher scores on average. There was no significant difference in change in naming vocabulary scores between children from stable income households and their peers from households who experienced downward (*β* = 0.028, *p* > 0.05) or fluctuating income (*β* = 0.008, *p* > 0.05). The association between age 3 and age 5 naming vocabulary scores indicates a high degree of stability in naming vocabulary scores over time (*β* = 0.518, *p* < 0.001).

**Table 3:** Standardized regression coefficients of income dynamics (average income and volatility) and change in cognitive ability from ages 3 to 5.

	Naming vocabulary age 5 *n* = 3,521		Picture similarity age 5 *n* = 3,518	
	*β*	SE	*β*	SE
Cognitive ability (Age 3)^1^	0.518	0.015^***^	0.281	0.022^***^
Average income (T1–T5)	0.081	0.025^***^	0.029	0.027
Income volatility (Ref: No Change)				
Upward (gaining)	0.036	0.016^*^	−0.012	0.017
Downward (losing)	0.028	0.018	−0.040	0.018^*^
Fluctuating (↓↑/↑↓)	0.008	0.016	−0.049	0.017^**^
*Control*				
Parental education				
Degree (Ref)				
Vocational	−0.047	0.021^*^	−0.034	0.028
High school	−0.063	0.024^**^	−0.033	0.029
No qualification	−0.096	0.023^***^	−0.047	0.027
Parental class				
Higher manag/admin/prof (Ref)				
Intermediate	0.013	0.018	−0.002	0.025
Routine/manual/No work	−0.024	0.022	−0.037	0.024
Neighborhood poverty				
SIMD 5 (Ref—least deprived)				
SIMD 4	0.012	0.021	0.006	0.023
SIMD 3	0.001	0.018	0.008	0.028
SIMD 2	0.008	0.020	−0.010	0.023
SIMD 1 (most deprived)	−0.015	0.022	−0.066	0.030
Child gender (male)	−0.025	0.017	0.021	0.018
Child age	0.012	0.016	0.013	0.018
Family type (single)	0.016	0.022	0.003	0.022
Mother age at birth of cohort child				
>30 (Ref)				
20–29	0.012	0.025	0.020	0.028
<20	−0.011	0.016	−0.007	0.019
*R* ^2^	0.34		0.12	

SIMD, Scottish Index of Multiple Deprivation; Higher manag/admin/prof, higher managerial, administrative, and professional occupations.

1 Either naming vocabulary or picture similarity score at age 3, respectively.

* *p* < 0.05;

** *p* < 0.01;

*** *p* < 0.001.

**Figure 2:**
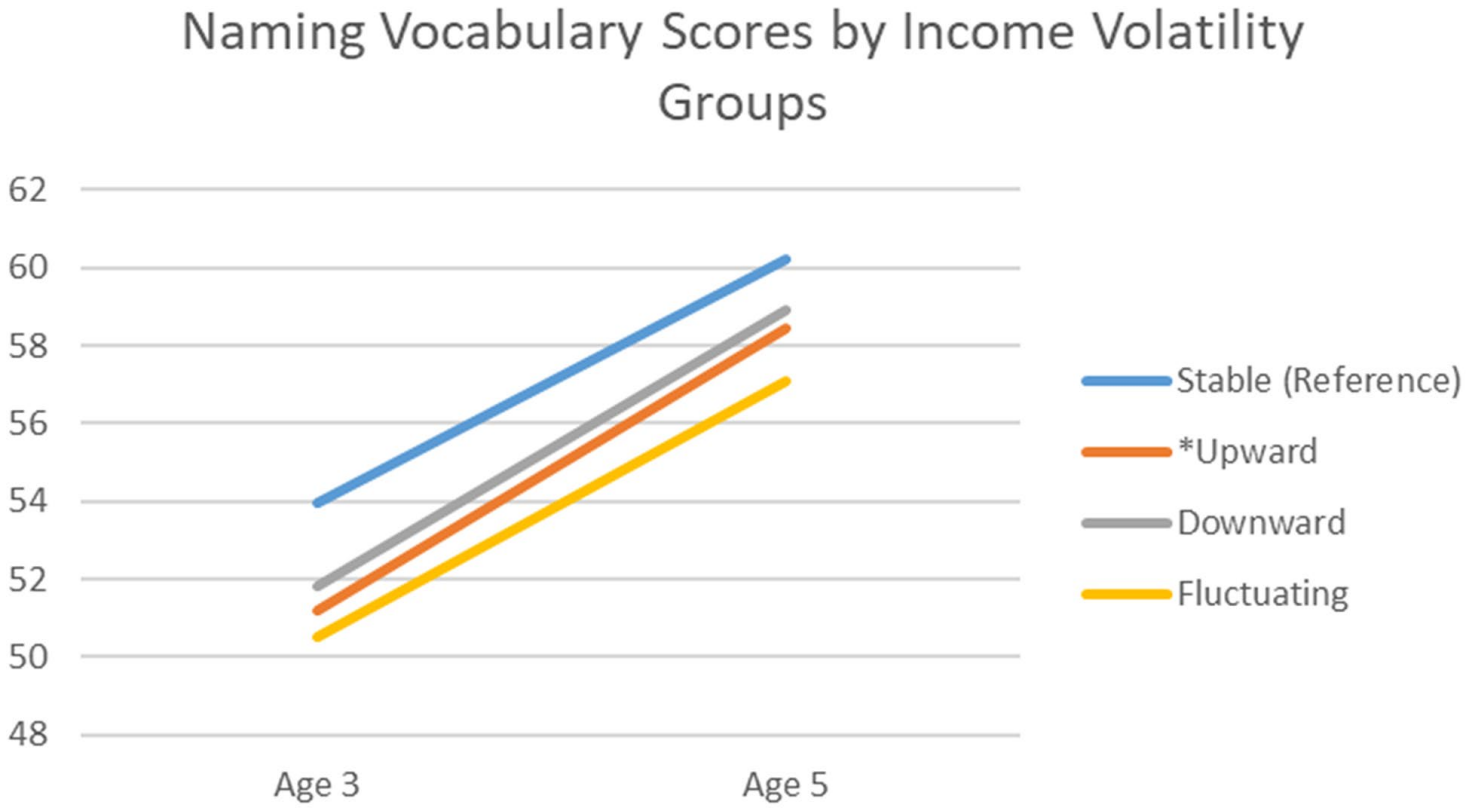
Trajectory of naming vocabulary scores from age 3 to 5 across income volatility groups. *Note:* ^*^Statistically significant change between volatility group and stable income group in the regression analysis.

Figure 2 is a descriptive graph showing trends in naming vocabulary scores for children from families experiencing different forms of income change. There was an increase in naming vocabulary scores for all children. However, children whose families experienced upward income change witnessed a significantly higher increase in scores (average of 7.24 points) than their peers from stable income families (average of 6.24 points).

Results for picture similarity indicate that both downward (*β* = −0.040, *p* < 0.05) and fluctuating income volatility (*β* = −0.049, *p* < 0.01) were negatively associated with change in picture similarity scores from ages 3 to 5. Specifically, compared to children from households with stable income, those whose households experienced an income loss and fluctuating income recorded significantly lower growth in problem-solving and reasoning scores. Neither average income (*β* = 0.029, *p* > 0.05) nor income gain (*β* = −0.012, *p* > 0.05) were significantly associated with change in picture similarity scores. There was a smaller degree of stability in picture similarity scores over time (*β* = 0.280, *p* < 0.001) compared to naming vocabulary scores.

**Figure 3:**
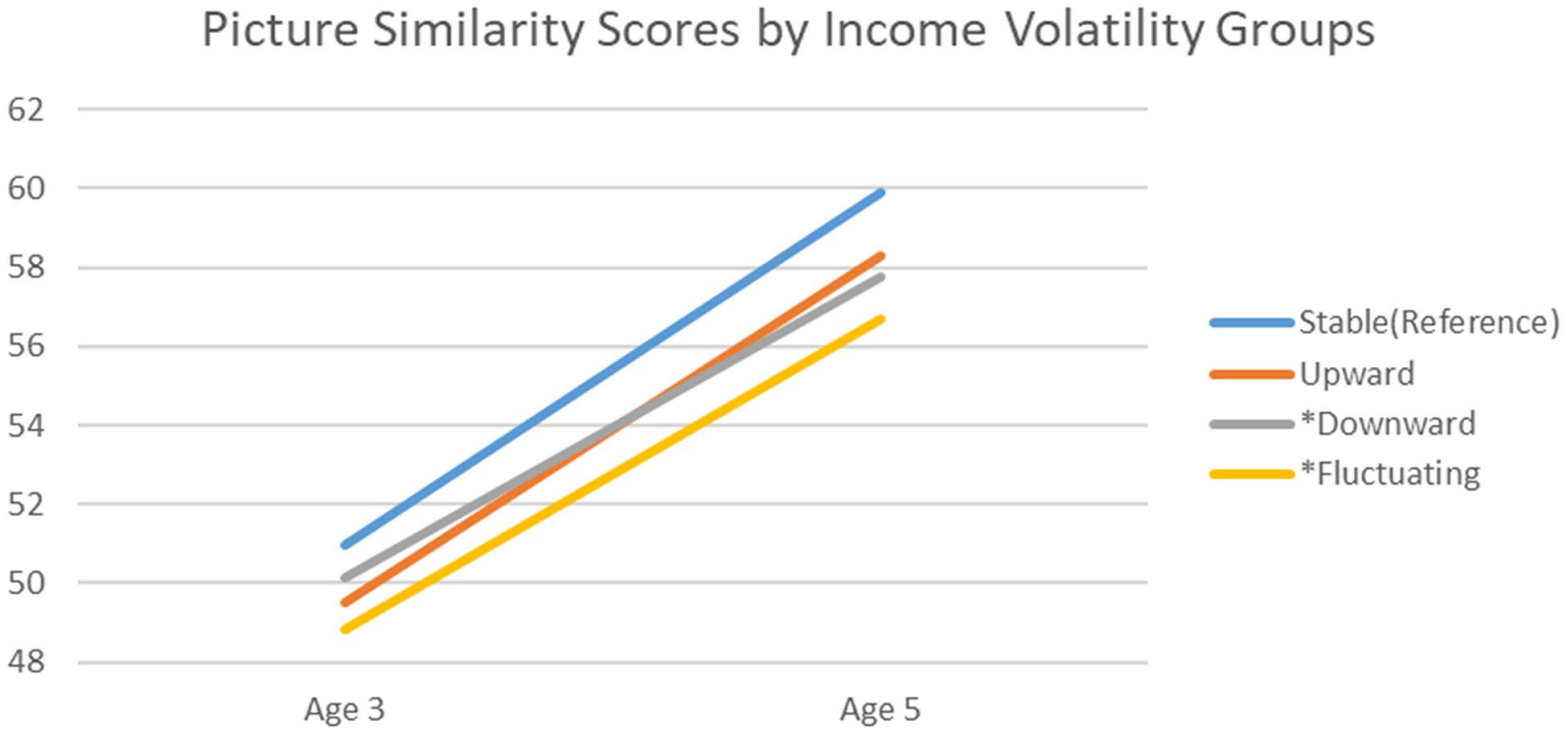
Trajectory of picture similarity scores from age 3 to 5 across income volatility groups. *Note:* ^*^Statistically significant change between volatility group and stable income group in the regression analysis.

Figure 3 is a descriptive graph showing trends in picture similarity scores for children from families experiencing different forms of income change. All children demonstrated a significant increase in problem-solving scores across time. However, children whose families experienced downward or fluctuating income witnessed a significantly lower increase in scores (average of 7.62 and 7.87 points, respectively) than their peers from stable income families (average of 8.92 points).

### Moderating Effect of Family Income Position on Volatility

Table 4 reports results examining whether the association between income volatility and cognitive development is moderated by a family’s overall income position. The log-likelihood test comparing constrained and unconstrained models indicates similar patterns of association between income volatility and naming vocabulary [*X^2^*(19) = 20.106, *p* > 0.05], and income volatility and picture similarity [*X^2^*(19) = 12.95, *p* > 0.05] for children from low and medium–high-income households.

**Table 4:** The standardized coefficients of income dynamics (average income and volatility) on change in cognitive ability from ages 3 to 5 for low and medium–high-income families.

	Naming vocabulary age 5				Picture similarity age 5			
	Low income (*n* = 747)		Medium–High income (*n* = 2,774)		Low income (*n* = 744)		Medium–High income (*n* = 2,774)	
	*β*	(SE)	*β*	(SE)	*β*	(SE)	*β*	(SE)
Cognitive ability (Age 3)^1^	0.507	0.021^***^	0.511	0.014^***^	0.302	0.024^***^	0.258	0.022^***^
Average income (T1–T5)	0.010	0.005	0.042	0.022	0.005	0.006	0.020	0.023
Income volatility (Ref: no change)								
Upward (gaining)	0.043	0.017^*^	0.036	0.014^*^	−0.012	0.020	−0.009	0.016
Downward (losing)	0.024	0.020	0.023	0.018	−0.043	0.019^*^	−0.040	0.017^*^
Fluctuating (↓↑/↑↓)	0.004	0.020	0.003	0.015	−0.058	0.021^**^	−0.041	0.015^**^
*Control*								
Parental education								
Degree (Ref)								
Vocational	−0.052	0.021^*^	−0.057	0.023^*^	−0.033	0.026	−0.036	0.029
High school	−0.072	0.027^**^	−0.070	0.025^**^	−0.039	0.030	−0.037	0.029
No qualification	−0.116	0.030^***^	−0.067	0.018^***^	−0.059	0.035	−0.034	0.020
Parental class								
Higher manag/admin/prof (Ref)								
Intermediate	0.007	0.015	0.010	0.020	−0.002	0.020	−0.002	0.026
Routine/manual/No work	−0.017	0.017	−0.023	0.023	−0.025	0.018	−0.034	0.024
Neighborhood poverty								
SIMD 5 (Ref—least deprived)								
SIMD 4	0.007	0.016	0.010	0.023	0.002	0.017	0.004	0.024
SIMD 3	0.002	0.017	0.002	0.019	0.008	0.025	0.009	0.028
SIMD 2	0.009	0.023	0.008	0.020	−0.011	0.026	−0.010	0.022
SIMD 1 (most deprived)	−0.021	0.026	−0.015	0.018	−0.081	0.035^*^	−0.056	0.025
Child gender (male)	−0.024	0.017	−0.024	0.017	0.019	0.019	0.019	0.019
Child age	0.020	0.018	0.018	0.016	0.011	0.020	0.010	0.017
Family type (single)	0.007	0.029	0.004	0.015	−0.003	0.028	−0.002	0.014
Mother age at birth of cohort child								
>30 (Ref)								
20–29	−0.008	0.016	−0.007	0.016	−0.003	0.020	−0.003	0.019
<20	0.019	0.037	0.008	0.016	0.026	0.042	0.011	0.018
*R^2^*	0.28		0.29		0.11		0.09	

SIMD, Scottish Index of Multiple Deprivation; Higher manag/admin/prof, higher managerial, administrative, and professional occupations.

1 Either naming vocabulary or picture similarity score at age 3, respectively. All similar models constrained to be equal across groups.

* *p* < 0.05;

** *p* < 0.01;

*** *p* < 0.001.

The findings were also similar to those found for the main model. Specifically, income gain was positively associated with changes in naming vocabulary outcomes from ages 3 to 5 for children from low (*β* = 0.043, *p* < 0.05) and medium–high-income families (*β* = 0.036, *p* < 0.01). Additionally, both downward (low income: *β* = −0.043, *p* < 0.05; medium–high income: *β* = −0.040, *p* < 0.05) and fluctuating income volatility (low income: *β* = −0.058, *p* < 0.01; medium–high income; *β* = −0.041, *p* < 0.01) were negatively associated with changes in picture similarity scores from ages 3 to 5.

### Sensitivity Analyses

To test the robustness of the findings, we performed all analyses again using only those participants with complete data on income measures across time (*n* = 2,869). There were no differences in the patterns of significance or direction of estimates for income, volatility, or covariates on change in cognitive ability measures (see Table 5).

**Table 5:** Standardized regression coefficients of income dynamics (average income and volatility) and change in cognitive ability from ages 3 to 5.

	Naming vocabulary age 5 *n* = 2,801		Picture similarity age 5 *n* = 2,800	
	*β*	SE	*β*	SE
Cognitive ability (Age 3)^1^	0.503	0.016^***^	0.278	0.025^***^
Average income (T1–T5)	0.094	0.029^**^	0.038	0.030
Income volatility (Ref: No Change)				
Upward (gaining)	0.037	0.019^*^	−0.022	0.019
Downward (losing)	0.016	0.021	−0.051	0.021^*^
Fluctuating (↓↑/↑↓)	0.021	0.018	−0.043	0.020^*^
*Control*				
Parental education				
Degree (Ref)				
Vocational	−0.051	0.024^*^	−0.035	0.030
High school	−0.076	0.027^**^	−0.037	0.030
No qualification	−0.076	0.026^**^	−0.024	0.026
Parental class				
Higher manag/admin/prof (Ref)				
Intermediate	0.016	0.020	0.002	0.024
Routine/manual/No work	−0.003	0.025	−0.021	0.023
Neighborhood poverty				
SIMD 5 (Ref—least deprived)				
SIMD 4	0.008	0.025	−0.005	0.023
SIMD 3	0.005	0.020	0.010	0.028
SIMD 2	−0.004	0.024	−0.011	0.024
SIMD 1 (most deprived)	−0.023	0.024	−0.064	0.030
Child gender (male)	−0.023	0.018	0.017	0.020
Child age	0.023	0.020	0.007	0.022
Family type (single)	0.017	0.025	0.012	0.026
Mother age at birth of cohort child				
>30 (Ref)				
20–29	−0.016	0.018	−0.021	0.020
<20	0.013	0.025	0.018	0.031
*R^2^*	0.32		0.12	

SIMD, Scottish Index of Multiple Deprivation; Higher manag/admin/prof, higher managerial, administrative and professional occupations.

1 Either naming vocabulary or picture similarity score at age 3, respectively.

* *p* < 0.05;

** *p* < 0.01;

*** *p* < 0.001.

## Discussion

In the current study, we investigated the associations between family income and volatility and child cognitive development in the early years. Additionally, we examined whether the association between income volatility and cognitive development are dependent on a family’s income position. As in previous studies (see e.g., Cooper and Stewart, 2021), children from households with higher average income recorded a significant increase in cognitive development than their peers from low-income households, albeit only for receptive vocabulary. Average family income was not significantly associated with changes in children’s problem-solving abilities.

Apart from average income, we found associations for upward, downward, and fluctuating volatility and changes in cognitive outcomes, indicating that volatility is an additional important dynamic of family income that influences cognitive development in the early years. Compared to children from households with stable income, children whose families experienced upward income demonstrated a higher increase in expressive vocabulary, although children in stable income families obtained the highest score. Conversely, children whose families experienced downward and fluctuating income recorded significant lower levels of change in their problem-solving abilities. While these findings are consistent with emerging evidence on the associations between volatility and childhood behavior outcomes (Miller and Votruba-Drzal, 2017), postsecondary educational outcomes (Hardy, 2014), adolescent school engagement (Gennetian et al., 2015), and school attendance (Gennetian et al., 2018), our study provides several new insights. Specifically, we show that forms of volatility vary in their degree of importance and may depend on the domain of development under consideration. Given that similar variabilities have been observed for the association between average family income and developmental outcomes (Cooper and Stewart, 2021), there is a need for nuanced theoretical conceptualizations that accounts for possible domain-specific differences in the association between income and child outcomes. Additionally, we found that familial experiences of fluctuating income are associated with child cognitive development, a category, which has hitherto not been examined (Hill, 2018).

Compared to average income, volatility in our study was consistently associated with changes in the examined domains of cognitive outcome. This contrasts with previous studies where average income levels were more strongly associated with outcomes of interest (Hardy, 2014; Miller and Votruba-Drzal, 2017). However, our study is different from these previous studies in that we investigated changes in developmental outcomes rather than associations between volatility and later outcomes (Hardy, 2014) or trajectories of these outcomes (Miller and Votruba-Drzal, 2017). When considering the domain where both average income and volatility were associated with cognitive outcome, average income had a stronger association with changes in expressive vocabulary. This, on the other hand, is consistent with the view that long-term family income position during a child’s formative years is a more important determinant for future developmental outcomes than short-term changes (Miller and Votruba-Drzal, 2017). This relative importance of income and volatility may also depend on the developmental domain of interest, an important dimension that should be examined by future studies.

Although research into the mechanisms underpinning the association between volatility and cognitive development is required, previous studies suggest higher income drives parental investment in educational resources (Akee et al., 2010; Duncan et al., 2011; Bastagli et al., 2018), improves parental psychological wellbeing, and positive parent–child interaction (Shaw and Shelleby, 2014). On the other hand, economic pressures negatively influence parental investment, psychological wellbeing, parent–child interaction, and child-level stress (Evans et al., 2007; Prause et al., 2009; Blair et al., 2011; Dahl et al., 2014; Cooper and Stewart, 2021). Based on existing evidence, we hypothesize that in the very early years, gains in income will lead to greater improvement in cognitive domains associated with language development due to ability of parents to invest in educational resources and positive parent–child interaction with these resources. On the other hand, income losses or fluctuations will stifle the development of problem-solving abilities, possibly through an increase in parental stress, negative parenting, and child-level stress.

A key question of interest in the present study was whether the association between income volatility and changes in child cognitive outcome is dependent on a family’s overall income position. Contrary to our theoretical hypothesis and previous empirical findings (Gennetian et al., 2015), we found similar patterns of associations between volatility and cognitive development for children living in poverty and children from medium–high-income homes. In all family groups, upward income was associated with a higher increase in children’s expressive vocabulary while downward and fluctuating income were associated with a lower growth in problem-solving abilities. However, our finding is similar to patterns observed in Hardy’s (2014) study, whereby the association between volatility and educational attainment was not statistically different between moderate- and low-income families. The sensitivity of middle–high-income families to downward and fluctuating volatility may be due to the likelihood of volatility reducing income for these families without the possibility of access to social support due to their overall income positions. Income swings among these families may therefore prevent them from maintaining existing levels of investment (Miller and Votruba-Drzal, 2017) relevant for child cognitive development. More nuanced policy interventions that consider swings in family income particularly for those living on medium income are required to support developmental outcomes for all children.

Compared to previous research on income volatility, we adopted an approach that enables us to contribute to the literature on income volatility and developmental outcomes in several ways. First, we directly modeled changes in cognitive outcomes by controlling for previous cognitive outcomes rather than simply relying on the temporal order in which income volatility and the developmental outcome of interest are measured. Second, while volatility simply defines swings in income, the direction of swing is important in determining effects on childhood outcomes. Previous studies have mainly examined directions of swings in the form of income gains or losses. What is new in our study is a third, previously unexamined category, fluctuating income, which represents either a gain followed by a loss, or vice versa (Hill, 2018). Overall, our approach enabled us to decouple the impact of different forms of volatility and provide new evidence that short-term volatility is associated with significant changes in child cognitive outcomes. Finally, our findings suggest a need for nuanced theoretical reconceptualization of the association between income and developmental outcomes to fully understand its multidimensional impact on life course outcomes. Specifically, existing theoretical perspectives (e.g., the family stress and investment models) should be expanded to incorporate other dynamics of income such as volatility, in addition to average family income. Further, theories need to take cognizant of the fact that the relative importance of various income dynamics as well as their mechanisms of effect may depend on the developmental outcome of interest.

### Limitations

The present findings should be understood within the context of specific limitations. First, the measure of income in our data set was based on self-reporting, which raises issues of reliability. However, benchmarks within the data, such as computations of poverty thresholds, suggest that this is consistent with national figures (Sosu and Schmidt, 2017). Second, we measured volatility over a short period rather than across all time points of data collection. Volatility measures based on a longer duration of income data might result in different outcomes. However, a unique aspect of the current study was the ability to examine associations between volatility and changes in cognitive outcomes.

### Policy and Research Implications

Our findings have important implications for improving child developmental outcomes. Policies that involve increases in family income and those designed to reduce negative income shock and fluctuations are likely to have immediate impact on children’s cognitive development as well as other developmental domains (Morris and Gennetian, 2003; Bastagli et al., 2018). Although there is availability of social support within the United Kingdom context to protect families against income volatility (Bartels and Bönke, 2013; Avram et al., 2019), this may not be enough to cushion the effects of downward and fluctuating income on children’s cognitive development. Evidence from both our study and previous findings suggests that volatility is more prevalent among low-income families (Hill, 2018), and these families generally have limited resources to draw on to cushion its impacts (Page et al., 2009). As a result, children from households living in poverty are more likely to experience the greatest benefits from policies aimed at increasing family income. For middle-income families, more nuanced policies are required to enable families who experience income volatility to gain social support and ensure stability in household earnings. We argue that these interventions will have a positive impact on child cognitive development and create multiple long-term benefits for all children, and they are likely to cost less than addressing the effects of poor educational outcomes in later life (Scott et al., 2001).

Our study is particularly timely given the current global context of the COVID-19 pandemic, which has resulted in rapid changes to family income around the world and is predicted to lead to increased volatility in family income in the coming years (Bourquin et al., 2020; Kansiime et al., 2021). The data we used for this study covers the period of the Great Recession of 2008–2012, which saw a rise in family volatility (Avram et al., 2019). Our findings, therefore, suggest a need for governments to develop robust policies that cushion families from rapid income changes in order to reduce the already devastating effect of the COVID-19 pandemic on children’s developmental outcomes arising from school closures (Engzell et al., 2021). Finally, the limited attention devoted to understanding the influence of income volatility in the developmental literature to date may have resulted in an underestimation of the total effect of the dynamics of income on children’s future outcomes. Future studies should therefore include a volatility measure in addition to average income to provide a more comprehensive understanding of the dynamics of income on child developmental outcomes.

## Conclusion

Understanding the nature of the relationship between family economic circumstances and childhood outcomes is crucial for improving life chances for children growing up in economic adversity. Our findings point out that other dynamics of income, such as upward, downward, and fluctuating volatility, influence children’s cognitive development over and above parental average income. Adopting a nuanced conceptualization of family income will help to determine its multidimensional effects on developmental outcomes and the types of policies needed to improve outcomes for all children in families facing economic hardship.

## Data Availability Statement

Publicly available datasets were analyzed in this study. This data can be found at: https://beta.ukdataservice.ac.uk/datacatalogue/series/series?id=200020.

## Ethics Statement

Ethical approval for the GUS study was received from the Scotland’s Scottish “A” Multicentre Research Ethics Committee (MREC; application reference: 04/M RE 1 0/59), and approval for the data use for this study was obtained through the United Kingdom Data Service. Written informed consent to participate in this study was provided by the participants’ legal guardian/next of kin.

## Author Contributions

ES and PS contributed to the conceptualization of the study, undertook data analysis, and contributed to the writing of the manuscript. ES undertook literature review. All authors contributed to the article and approved the submitted version.

## Funding

The work was supported by the British Academy Skills acquisition award SQ120023.

## Conflict of Interest

The authors declare that the research was conducted in the absence of any commercial or financial relationships that could be construed as a potential conflict of interest.

## Publisher’s Note

All claims expressed in this article are solely those of the authors and do not necessarily represent those of their affiliated organizations, or those of the publisher, the editors and the reviewers. Any product that may be evaluated in this article, or claim that may be made by its manufacturer, is not guaranteed or endorsed by the publisher.
